# Management of an extended clivus fracture: a case report

**DOI:** 10.1186/1756-0500-6-554

**Published:** 2013-12-23

**Authors:** Julia JE Evers, Volker VV Vieth, René RH Hartensuer, Michael MJR Raschke, Thomas TV Vordemvenne

**Affiliations:** 1 Department for Trauma-, Reconstructive- and Handsurgery, University Muenster, Albert-Schweitzer-Campus 1 Gebäude W1, Münster 48149, Germany; 2 Department for Clinical Radiology, University Muenster, Albert-Schweitzer-Campus 1 Gebäude W1, Münster 48149, Germany

**Keywords:** Clivus fracture, Craniocervical junction, Halo device

## Abstract

**Background:**

Clivus fractures are highly uncommon. The classification by Corradino *et al*. divides the different lesions in longitudinal, transverse and oblique fractures. Longitudinal types are associated with the highest mortality rate between 67 – 80%. Clivus fractures are often found after high velocity trauma, especially traffic accidents and falls. The risk of neurologic lesions is high, because of the anatomic proximity to neurovascular structures like the brainstem, the vertebrobasilar artery, and the cranial nerves. Longitudinal clivus fractures have a special risk of causing entrapment of the basilar artery and thus ischemia of the brainstem.

**Case presentation:**

This lesion in our patient was a combination-fracture of the craniocervical junction with a transverse clivus fracture. In this case, the primary closed reduction of the clivus fracture and the immobilization with a halo device was the therapy of choice and led to consolidation of the fracture.

**Conclusion:**

Therapy advices and examples in the literature are scarce. We present a patient with a clivus fracture, who could be well treated by a halo device. Through detailed research of the literature a therapy algorithm has been developed.

## Background

Clivus fractures are highly uncommon [1,2]. The classification by Corradino *et al.*[2] divides the different lesions in longitudinal, transverse and oblique fractures. Longitudinal types are associated with the highest mortality rate between 67 – 80% [2]. Clivus fractures are often found after high velocity trauma, especially traffic accidents and falls [3,4]. The risk of neurologic lesions is high, because of the anatomic proximity to neurovascular structures like the brainstem, the vertebrobasilar artery, and the cranial nerves. Longitudinal clivus fractures have a special risk of causing entrapment of the basilar artery and thus ischemia of the brainstem [3].

## Case presentation

A 43-year-old polytraumatized male patient was admitted in May 2009 to our clinic. He had fallen off a scaffolding, which was 4 meters high. Initially he showed movement of all four extremities and was stable from respiratory and hemodynamic point of view. However, due to a deteriorated mental state the patient was intubated at the scene of the accident.

**Figure 1:**
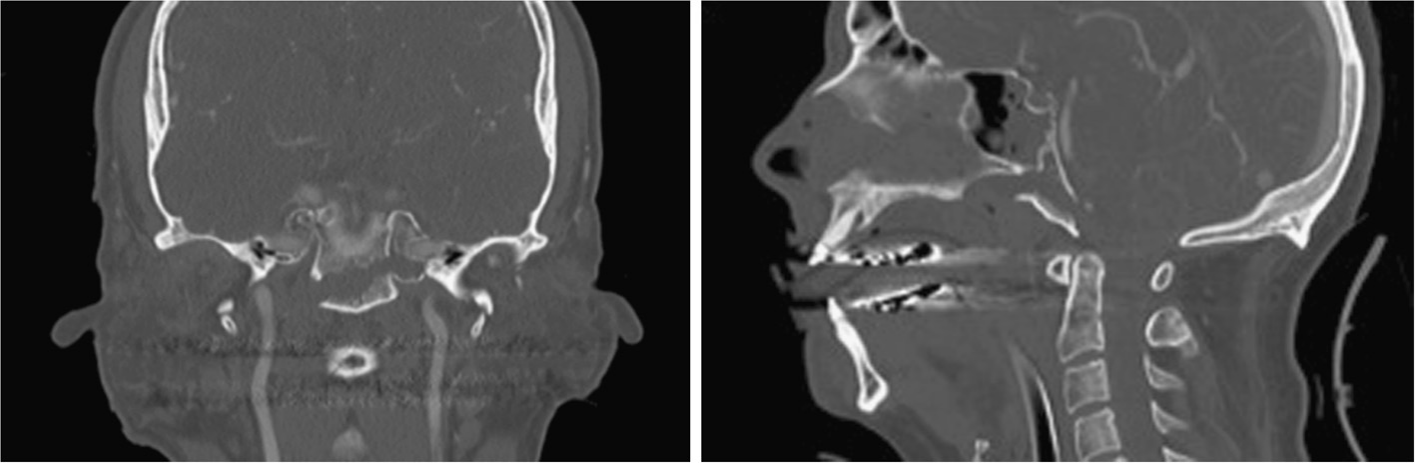
antero-posterior and lateral view of clivus fracture on the day of admission.

**Figure 2:**
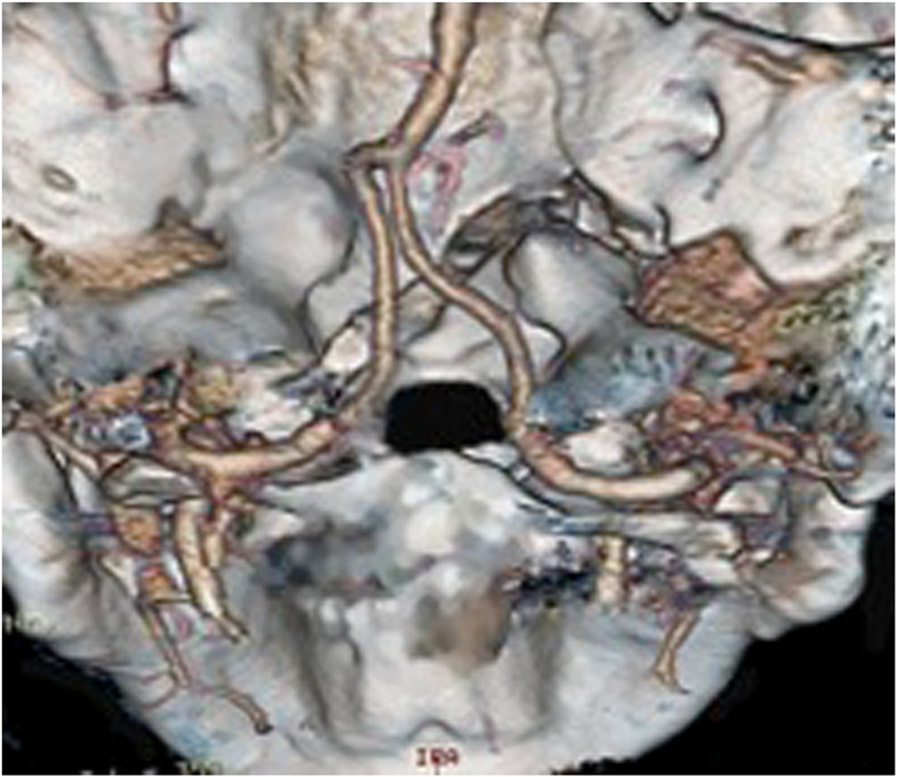
Computed tomography reconstruction of the craniocervical conjunction with fracture extending into the left occipital condyle.

**Figure 3:**
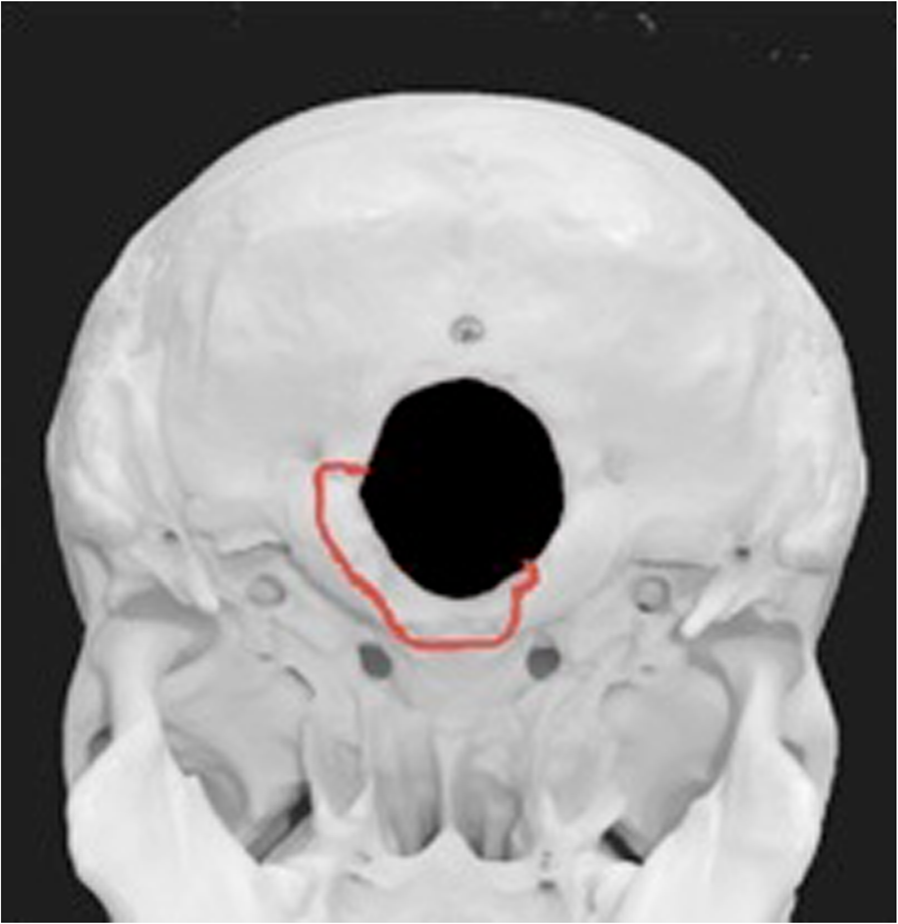
Schematic depiction of the fracture’s course.

The computed tomography (CT) scan revealed the following injuries:

• Dislocated clivus fracture – transverse type [2], extending into the left occipital condyle (Figure 1, Figure 2, Figure 3)

• Fracture of the atlas, anterior arch

• Fractures of the transverse processes of the lumbar vertebral bodies 1 to 4 and thoracic vertebral bodies 5 to 10

• Traumatic brain injury with subarachnoidal bleeding manifested by initial unconsciousness and retrograde amnesia

• Pelvic fractures with non-dislocated fractures of the anterior and posterior columns of the acetabulum, as well as the os ilium, and transforaminal fracture of the sacrum

• Fracture of the left clavicle

• Multiple rib fractures, left thorax

• Subluxation of the jaw-joint

**Figure 4:**
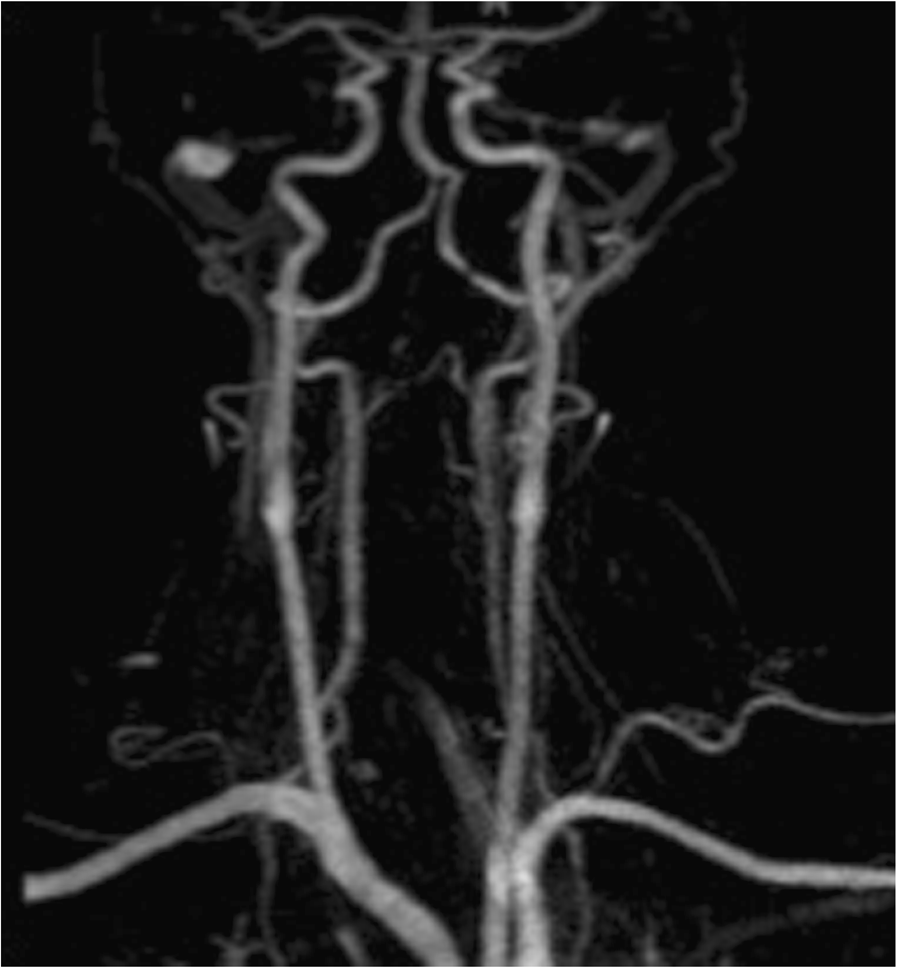
Magnetic resonance imaging angiography of the cervical vessels.

**Figure 5:**
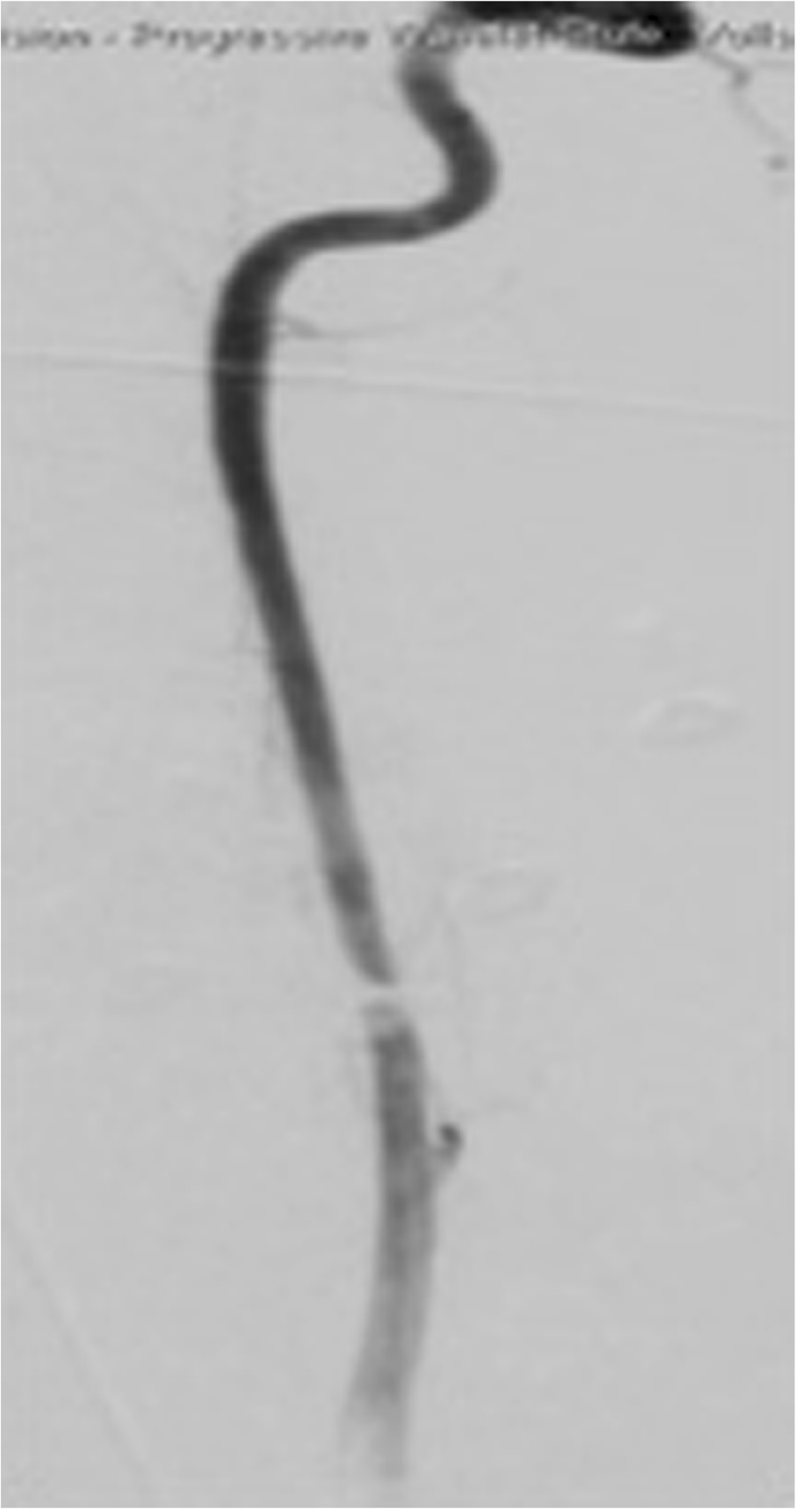
Angiography with proof of dissection of the left vertebral artery in segment V4.

**Figure 6:**
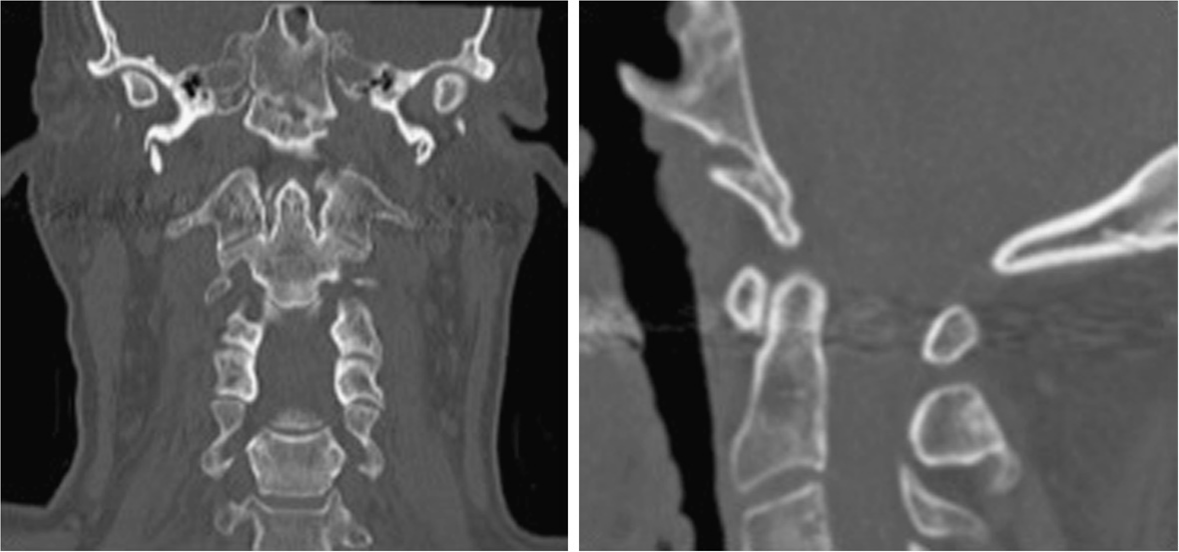
Computed tomography of the cervical spine after 12 weeks of immobilization with a halo-device, proving the consolidation of the fracture.

**Figure 7:**
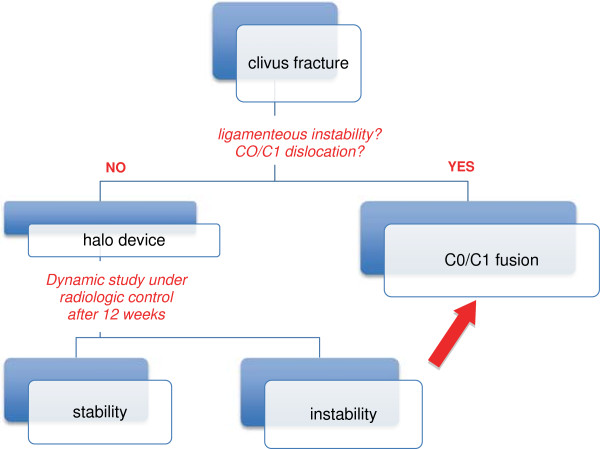
Diagram 1: Algorithm for the management of craniocervical lesions.

The Injury Severity Score (ISS) was 41. After the initial diagnostics, the patient was brought to the operating theatre, where a closed reduction of the clivus fracture under manual traction and c-arm control was performed. A primary immobilization of the cervical spine was achieved by use of a halo device. According to the guidelines [5] a CT-angiography of the cervical vessels was performed. As a result of this routine imaging, suspicion of a dissection of the left vertebral artery in segment V4 was entertained. Anticoagulation with heparin was started. The following angiographic magnetic resonance imaging (MRI) and conventional 4-vessel-angiography confirmed the initial suspicion (Figures 4 and 5). In the MRI of the cervical spine no further ligamentous instability could be detected. Due to the complex injuries sustained, the road to recovery for the patient after intensive care treatment was difficult, but ultimately successful. After 12 weeks of immobilization the follow-up CT-scan showed a sufficient bony bridging of the clivus fracture and the halo fixation was removed Figure 6. The ligamentous stability of the cranial-cervical junction was proved with a dynamic examination of the cervical spine under radiologic C-arm control. The concluding neurologic examination showed no abnormalities of the cranial nerves. A mild motor deficit was detected in the left arm and attributed to a lesion of the left brachial plexus. A mild deficit was also found in the left leg and was considered a result of trauma to the head. A final conventional angiography of the vertebral artery could no longer detect a dissection and the anticoagulation was stopped 6 months after trauma. After rehabilitation the patient was re-integrated back into his trained profession Figure 7.

## Discussion

The risk for patients with a clivus fracture of sustaining concomitant neurovascular deficiencies is high because of the anatomic proximity to cranial nerves, the brainstem and the vertebrobasilar artery. With longitudinal clivus fractures especially, an entrapment of the basilar artery can occur and lead to ischemia of the brainstem, which may result in death of the patient. There are also reports in the literature, however, of possible preventions of this fatal course. Sato *et al.*[6] treated patients with a concomitant vascular lesion with aspirin 100 mg per day. Another treatment option, found in the literature, was anticoagulation with argatroban [7]. Further patients could successfully be treated with anticoagulation through heparin and later warfarin [5].

There are also descriptions in the literature of lethal courses of concomitant vascular injuries such as a report of death of a patient with recurrent vasospasms of the carotid artery [3]. Further, clivus fractures with traumatic brain injuries are associated with very high risk of life-threatening complications [3,6]. But in many cases, neurologic deficits secondary to lesions of the cranial nerves can be found. Commonly the 6th and 7th cranial nerves are affected. Whereas a 6th nerve palsy is often linked to transverse clivus fractures, a 7th is usually affected only in longitudinal fracture types [3,6,8,9]. Further symptoms that may also occur are rhinorrhea, otorrhea [3,9], fistulas between the carotid artery and the sinus cavernosus, or an ophthalmoplegia [10].

With extension into the petrous bone, a deterioration of hearing may occur [5,8]. In the case of a lesion of the sella turcica, diabetes insipidus may result, which in most cases is transient and can be treated by a temporary hormone substitution [3].

The list of possible symptom complexes is long and well described in the literature. But options for a feasible therapy for this fracture entity are scarce. Dashti *et al.*[4] report a successful course of a patient with a clivus fracture, treated with a halo.

A more unstable injury of the craniocervical conjunction like reported by Maughan *et al.*[11] with an avulsion fracture of the foramen magnum with bilateral fractures of the occipital condyles, and extension into the inferior clivus attained good results with an occipito-cervical fusion.

This fracture in our patient was a combination-fracture of the craniocervical junction. Due to the extension of the clivus fracture into the left occipital condyle, exact classification of this fracture type was difficult. The anterior arch fracture of the atlas itself was stable and did not need as rigid a stabilization as the halo device.

The extension of the fracture line into the occipital condyle alone was comparable to a type II fracture according to Anderson and Montesano [12]. This fracture entity was regarded as stable or unstable, depending on radiologic signs for instability and or ligamentous disruption and should be treated with or a hard collar or a halo device or a surgical stabilization [13].

As this was a complex craniocervical lesion, with an extended fracture of the clivus together with its complete avulsion and associated disruption of the alar ligaments, the decision to apply a halo device was made.

In this case, the primary closed reduction of the clivus fracture and the immobilization with a halo device was the therapy of choice and led to consolidation of the fracture. A final dynamic examination of the cervical spine after removal of the halo was conducted to exclude persistent ligamentous instability. Persistent ligamentous instability would indicate an occipito-cervical fusion.

## Conclusion

Clivus fractures are seldom seen. Therapy advices and examples in the literature are scarce. The CT-scan and the diagnostic investigation of the cervical vessels are the basics for the assessment of concomitant injuries. The treatment of choice is the closed reduction and immobilization of the cervical spine with a halo fixation. After immobilization a dynamic study of the cervical spine should be conducted to exclude persistent ligamentous instability. In this case, an occipito-cervical fusion must be performed.

## Consent

Written informed consent was obtained from the patient for publication of this Case Report and any accompanying images. A copy of the written consent is available for review by the Editor-in-Chief of this journal.

## Competing interests

The authors declare that they have no competing interests.

## Authors’ contributions

JE investigated on the patient’s data and drafted the manuscript. VV analyzed the radiologic data and made important contribution to the manuscript. RH participated in the design of the manuscript. MJR made important contribution in revision of the manuscript. TV participated in the investigation of the patient and analysis of the patient’s data. All authors read and approved the final manuscript.

## References

[B1] Ochalski GP Spiro RM Fabio A Kassam AB Okonkwo DO Fractures of the clivus: a contemporary series in the computed tomography era Neurosurgery 2009 6 6 1063 1069 10.1227/01.NEU.0000360154.18604.28 19934965

[B2] Corradino G Wolf AL Mirvis S Joslyn J Fractures of the clivus: classification and clinical features Neurosurgery 1990 6 4 592 596 10.1227/00006123-199010000-00015 2234364

[B3] Menkü A Koc RK Tucer B Durak AC Akdemir H Clivus fractures: clinical presentations and courses Neurosurg Rev 2004 6 3 194 198 15034764 10.1007/s10143-004-0320-2

[B4] Dashti R Ulu MO Albayram S Aydin S Ulusoy L Hanci M Concomitant fracture of bilateral occipital condyle and inferior clivus: what is the mechanism of injury? Eur Spine J 2007 6 Suppl 3 261 264 17180399 10.1007/s00586-006-0270-1 PMC2148078

[B5] Löhrer L Vieth V Nassenstein I Hartensuer R Niederstadt T Raschke MJ Vordemvenne T Blunt cerebrovascular injuries in acute trauma care: a screening protocol Eur Spine J 2012 6 5 837 843 10.1007/s00586-011-2009-x 21898164 PMC3337903

[B6] Sato S Iida H Hirayama H Endo M Ohwada M Fujii K Traumatic basilar artery occlusion caused by a fracture of the clivus--case report Neurol Med Chir 2001 6 541 544 10.2176/nmc.41.541 11758706

[B7] Taguchi Y Matsuzawa M Morishima H Ono H Oshima K Hayakawa M Incarceration of the basilar artery in a longitudinal fracture of the clivus: case report and literature review J Trauma 2000 6 6 1148 1152 10.1097/00005373-200006000-00023 10866264

[B8] Bala A Knuckey N Wong G Lee GYF Longitudinal clivus fracture associated with trapped basilar artery: unusual survival with good neurological recovery J Clin Neurosci 2004 6 6 660 662 10.1016/j.jocn.2003.11.008 15261246

[B9] Bonilha L Fernandes YB Mattos JPV Borges WAA Borges G Bilateral internuclear ophthalmoplegia and clivus fracture following head injury: case report Arq Neuropsiquiatr 2002 6 3-A 636 638 12244406 10.1590/s0004-282x2002000400023

[B10] Katsuno M Yokota H Yamamoto Y Teramoto A Bilateral traumatic abducens nerve palsy associated with skull base fracture--case report Neurol Med Chir (Tokyo) 2007 6 7 307 309 10.2176/nmc.47.307 17652916

[B11] Maughan PH Horn EM Theodore N Feiz-Erfan I Sonntag VKH Avulsion fracture of the foramen magnum treated with occiput-to-c1 fusion: technical case report Neurosurgery 2005 6 3 E600 10.1227/01.NEU.0000170989.90325.04 16145511

[B12] Anderson PA Montesano PX Morphology and treatment of occipital condyle fractures Spine (Phila Pa 1976) 1988 6 7 731 736 10.1097/00007632-198807000-00004 3194779

[B13] Karam YR Traynelis VC Occipital condyle fractures Neurosurgery 2010 6 3 Suppl 56 59 20173528 10.1227/01.NEU.0000365751.84075.66

